# Clinical Adjuvant Combinations Stimulate Potent B-Cell Responses *In Vitro* by Activating Dermal Dendritic Cells

**DOI:** 10.1371/journal.pone.0063785

**Published:** 2013-05-20

**Authors:** Katie Matthews, Nancy P. Y. Chung, Per Johan Klasse, Magda Moutaftsi, Darrick Carter, André M. Salazar, Steven G. Reed, Rogier W. Sanders, John P. Moore

**Affiliations:** 1 Department of Microbiology and Immunology, Weill Cornell Medical College, New York, New York, United States of America; 2 HIV Vaccine Initiative at Bill and Melinda Gates Foundation, Seattle, Washington, United States of America; 3 Infectious Diseases Research Institute (IDRI), Seattle, Washington, United States of America; 4 Oncovir, Inc., Washington D.C., United States of America; 5 Laboratory for Experimental Virology, Department of Medical Microbiology, Academic Medical Center, University of Amsterdam, Amsterdam, The Netherlands; University of California San Francisco, United States of America

## Abstract

CD14^+^ dermal DCs (CD14^+^ DDCs) have a natural capacity to activate naïve B-cells. Targeting CD14^+^ DDCs is therefore a rational approach for vaccination strategies aimed at improving humoral responses towards poorly immunogenic antigens, for example, HIV-1 envelope glycoproteins (Env). Here, we show that two clinically relevant TLR ligand combinations, Hiltonol plus Resiquimod and Glucopyranosyl lipid A plus Resiquimod, potently activate CD14^+^ DDCs, as shown by enhanced expression of multiple cytokines (IL-6, IL-10, IL-12p40 and TNF-α). Furthermore, the responses of CD14^+^ DDCs to these TLR ligands were not compromised by the presence of HIV-1 gp120, which can drive immunosuppressive effects *in vitro* and *in vivo*. The above TLR ligand pairs were better than the individual agents at boosting the inherent capacity of CD14^+^ DDCs to induce naïve B-cells to proliferate and differentiate into CD27^+^ CD38^+^ B-cells that secrete high levels of immunoglobulins. CD14^+^ DDCs stimulated by these TLR ligand combinations also promoted the differentiation of Th1 (IFN-γ-secreting), but not Th17, CD4^+^ T-cells. These observations may help to identify adjuvant strategies aimed at inducing better antibody responses to vaccine antigens, including, but not limited to HIV-1 Env.

## Introduction

Vaccines intended to counter some of the most globally significant pathogens (HIV-1, tuberculosis and malaria) are likely to require both humoral and T-cell responses, particularly Th1-mediated immunity. Subunit vaccines based on pathogen proteins offer safety and production advantages but require adjuvants to enhance their immunogenicity. Until recently, alum salts were the only adjuvants approved for human vaccines in the USA. Although alum is effective at boosting antibody (Ab) responses, the overall responses tend to be Th2-biased. Identifying adjuvant combination(s) capable of eliciting potent Th1 and Ab responses is a major challenge for developing vaccines against various infectious agents, including HIV-1.

Adjuvants generally activate the innate immune system by inducing inflammation at the administration site [1]. Central to this process are DCs, the most potent cells for initiating acquired immunity [2]. DCs express many innate immune receptors, including TLRs, that recognize conserved pathogen-associated molecular patterns, triggering DCs to become activated into mature cells [3]. The maturation process involves enhanced migration of DCs to draining lymphoid organs, up-regulation of MHC antigens and co-stimulatory molecules to drive the priming of naïve T-cells, and cytokine secretion to further polarize CD4^+^ T-cells.

TLR ligands belong to a new generation of adjuvants. Each TLR is triggered by a distinct set of microbial products; for example, TLR3 responds to double stranded RNA (mimicked by Polyinosinic: Polycytidylic acid (Poly(I:C)), TLR4 to lipopolysaccharide (LPS), and TLRs 7 and 8 to stimulatory single-stranded RNA (mimicked by Resiquimod (R-848)). The coupling of TLRs to signal transduction pathways is, except for TLR3, mediated via the MyD88 adaptor protein; TLR3 (and also TLR4) link to the adaptor protein TRIF. Of note is that MyD88 and TRIF act synergistically to fully activate DCs [4], [5].

The first TLR ligand approved for human use was monophosphoryl lipid A (MPL®), a non-toxic derivative of LPS [6]. MPL® combined with alum (AS04) is the adjuvant contained in two approved vaccines, Cervarix®, and Fendrix®, for human papilloma virus and hepatitis B virus, respectively. New generations of TLR4 agonists, including Glucopyranosyl lipid A (GLA), are more receptor-specific and induce fewer side effects [7]. GLA can be chemically modified to further enhance its biological activity, and has a good safety profile when combined with the Fluzone vaccine in monkey studies [8]. Although the vulnerability of Poly(I:C) to serum RNases limits its clinical utility, a more stable version, Hiltonol (Poly(I:CLC); Poly(I:C) and poly-L-lysine), is in late-stage clinical trials [1].

Immunizing via the skin is a rational approach since this organ contains abundant DCs, including Langerhans’ cells (LCs) and several dermal DC subsets. Intradermal vaccination may be superior to the clinically used subcutaneous and intramuscular routes [9], [10]. Each skin DC subset is equipped with an inherent specialized functionality [11]. Thus, LCs are potent stimulators of CTLs, while CD14^+^ dermal DCs (CD14^+^ DDCs) have a similar effect on naïve B-cells [12], [13]. Targeting CD14^+^ DDCs with TLR ligands is, therefore, a relevant strategy for improving Ab responses to vaccine antigens.

We have shown that both Poly(I:C) and LPS synergize with R-848 in activating CD14^+^ DDCs with potent B-cell and Th1 cell stimulatory capacity [14]. Here, we have extended that observation by using clinically relevant adjuvants (Hiltonol or GLA, combined with R-848). Moreover, we have assessed how these adjuvant combinations are affected by the HIV-1 envelope gp120 *in vitro*, a protein that, when administered as a vaccine in alum, elicits only weak and transient Ab responses [15], [16]. The limited immunogenicity is in part due to epitope shielding by glycans, but gp120 may also impair various immune cell functions, for example via binding to the Mannose-binding C-type lectin receptors (MCLRs), DC-SIGN and BDCA-2, on monocyte-derived DCs and plasmacytoid DCs, respectively [17]–[19]. There is evidence that gp120 can suppress immune responses to other antigens *in vivo* [20]–[22]. Although the mechanisms underlying any immunosuppressive effects operating *in vivo* are unknown, the abundant oligomannose glycans on gp120 may contribute [23], [24].

Here, we show that the Hiltonol plus R-848 and GLA plus R-848 TLR ligand combinations strongly activate human CD14^+^ DDCs to secrete high levels of B-cell and CD4^+^ T-cell stimulatory cytokines *in vitro*. Neither combination was compromised by the presence of gp120. Furthermore, the TLR-activated CD14^+^ DDCs both activated naïve B-cells and induced Th1 differentiation. Targeting CD14^+^ DDCs with either of these adjuvant combinations may, therefore, be suitable for an intradermal vaccination strategy aimed at inducing both Ab and Th1 responses to HIV-1 Env.

## Materials and Methods

### Ethics Statement

Skin samples were obtained from the New York Firefighters’ skin bank, New York Presbyterian Hospital. Skin was removed from cadaveric donors within 12 h post mortem (median age 48 years; range, 21–69 years). Written informed consent was obtained from all participants’ next of kin. Buffy coats were obtained from the New York Blood Center. We have exemption status from the Institutional Review Board for human blood products (exemption number: EXE 2008-016).

### Isolation of Cutaneous DC Subsets

Skin DCs were isolated as described previously [14]. Briefly, CD14^+^ DDCs and CD1a^+^ Migratory DCs (MiDCs) were isolated from skin migratory cells by positive selection using CD14 and CD1a microbeads (Miltenyi Biotec), respectively. Tissue-resident LCs were isolated from epidermal single-cell suspensions using CD1a microbeads following 1 h treatment of skin with dispase (2.4 U/ml; Invitrogen) at 37°C. Tissue-derived CD1a^+^ DDCs were isolated using CD1a microbeads following digestion of dermal sheets for 12 h with 0.2% collagenase [14]. In experiments involving TLR mRNA expression, Fluorescent activated cell sorting (FACS) was used to obtain highly purified epidermal LCs and dermal-derived CD1a^+^ and CD14^+^ DCs. Briefly, single-cell suspensions were overlayed onto Ficoll and centrifuged at 400 xg for 30 minutes. Cells were washed in PBS containing 10% FBS and blocked on ice for 30 minutes with human serum. Epidermal cells were stained with the following mAbs: anti-CD1a FITC (clone HI149), anti-CD45 APC (clone 2D1), anti-CD207 PE (clone DCGM4) and anti-HLA-DR APC-Cy7 (clone L243). Live LCs were identified as 7-AAD^−^ CD45^+^ HLA-DR^+^ CD1a^+^ CD207^+^ cells. Dermal-derived DCs were purified by FACS as described previously [14]. Briefly, live CD1a^+^ DDCs were identified as 7-AAD^−^ CD45^+^ HLA-DR^+^ CD1a^+^ CD14^−^ cells. Live CD14^+^ DDCs were identified as live 7-AAD^−^ CD45^+^ HLA-DR^+^ CD14^+^ CD1a^−^ CD1c^+^ SSC^lo^ cells.

### Injection of Human Skin

Human skin was sectioned into 5×5 cm explants, which were injected five times intradermally with 50 µl of PBS containing either 20 µg of Hiltonol, 5 µg of R-848, or both TLR activators (20 µg +5 µg). Controls consisted of explants injected with PBS only. Insulin needles (0.5 mm×16 mm Microlance; BD Biosciences) were used. Depending on the experiment, 3–10 explants per test group were collected. Immediately after injection, skin was rinsed in PBS containing antibiotics to remove any residual (non-injected) adjuvant(s). The explants were cultured, epidermal side-up, in Petri dishes for 48 h. Cell-free supernatants were collected and screened for cytokines by ELISA, and migratory cells were analyzed for surface markers.

### Cutaneous DC Stimulation

TLR ligands were obtained from Oncovir (Hiltonol), Infectious Diseases Research Institute (IDRI) (GLA) and Invivogen (R-848). Unless otherwise stated, they were used at the following concentrations: Hiltonol (10 µg/ml), GLA (500 ng/ml), R-848 (2.5 µg/ml). DCs (∼2×10^4^ cells) were stimulated in 96-well plates in a final volume of 250 µl. In some experiments, CD14^+^ DDCs were incubated for 1 h at 37°C with 1–10 µg/ml (see text) purified, endotoxin-free recombinant HIV-1 gp120 (JR-FL, clade B; Progenics Pharmaceuticals). TLR ligand(s) were then added for 48 h. Culture supernatants were screened for IL-6, IL-10, IL-12p40 and TNF-α expression using commercially available kits (BD Biosciences). Absorbances were measured at 450 nm (Molecular Devices reader). Data are presented as means ± SEM of values from duplicate wells. Quantification of BAFF, TLR3, TLR4, TLR7 and TLR8 mRNA was carried out as described previously [14].

### CD14^+^ DDC and Naïve B-cell Co-cultures

Naïve B-cells were purified to >95% by negative selection (Miltenyi Biotec) from peripheral blood mononuclear cells, as described previously [14]. B-cells were directly added (2×10^4^ cells) to CD14^+^ DDCs (DDC: B-cell ratios of 1∶5 and 1∶25) and cultured in the continuous presence of TLR ligands. For comparison, naïve B-cells were stimulated with the same TLR ligands, without DDCs. All cultures were supplemented with CD40L (200 ng/ml; Enzo Laboratories) and IL-2 (20 U/ml; Roche) [14]. For analysis of Ig secretion, B-cells were co-cultured with CD14^+^ DDCs for 14 d and supernatants screened by ELISA (Bethyl Laboratories).

### Mixed Lymphocyte Reaction (MLR)

Naïve CD4^+^ T-cells were purified to >95% by negative selection (Miltenyi Biotec). CD14^+^ DDCs were stimulated for 24 h prior to the addition of naïve CD4^+^ T-cells (T-cell to DDC ratio, 25∶1). After 6 days of co-culture, MLR supernatants were removed and IFN-γ (BD) and IL-21 (eBioscience) screened by ELISA. Intracellular cytokine staining was carried out following restimulation of T-cells with 100 ng/ml PMA (Phorbol 12-myristate 13-acetate) and 1 µg/ml ionomycin in the presence of Brefeldin A (1 µg/ml), as described previously [14].

### Proliferation Assays

B-cell proliferation was assessed using the BrdU cell proliferation ELISA according to the manufacturer’s instructions (Roche). Briefly, after a 5-day co-culture with DC subsets, cells were transferred to 96-well flat-bottomed plates and pulsed for 5h with 10 µM BrdU. Absorbance was measured at 450 nm, with data presented as mean O.D. values (± SEM) from triplicate wells.

### Phenotypic Analysis of DCs and B-cells

Skin DC subsets were analyzed by multicolor flow cytometry by staining with the following fluorochrome-labeled monoclonal Abs (mAbs): anti-CD1a FITC (clone HI149), anti-CD14 PE-Cy7 (clone 61D3), anti-CD40 APC (clone 5C3), anti-CD80 PE (clone L307.4), anti-CD86 PE (clone IT2.2), anti-CD184 APC (clone 12G5), anti-CD195 FITC (clone 2D7), anti-CD206 FITC (clone 19.2), anti-CD209 PE (clone eB-h209) and HLA-DR PerCP (clone L243). B-cells were analyzed using the following mAbs: anti-CD20 PE (clone 2H7), anti-CD27 PE-Cy7 (clone O323), anti-CD38 APC (clone HIT2) and anti-HLA-DR PerCP (clone L243). MAbs and relevant isotype controls were added for 30 min on ice. Cells were washed in PBS containing 2.5% FBS, fixed in 1% paraformaldehyde and analyzed with the LSR II cytometer (BD Biosciences) and Flowjo™ software.

### Statistical Analysis

Data are presented as the means ± SEM of independent experiments. We used nonparametric statistics, as too few data points were available to allow testing for Gaussian distribution. Skin-donor and other inter-experimental variation meant that pairing of the data points might be effective. When pairing was effective, we performed Wilcoxon matched-pairs test. In those cases, the Spearman rank coefficient, *r*, was in the range 0.6–0.9 and the pairing *p* value was 0.0004–0.03. In all other cases, we used the Mann-Whitney U test. One-tailed tests were used in order to evaluate the significance of the selected differences. Statistical analysis was performed using Prism Version 5 (GraphPad) software. A p value<0.05 was considered significant.

The possible synergistic effects of TLR ligands were analyzed as previously described [14]. We calculated the ratio between the highest cytokine response elicited by the combined stimuli and the sum of the responses to the two individual agents at their constituent concentrations. In cases where a single agent did not induce a detectable level of cytokine expression, the detection limit of the ELISA was used to calculate the sum of the responses to the two individual agents. These ratios were based on repeat measurements of each response with little to no variation, although there were too few replicates to allow a statistical evaluation. A ratio >1 is indicative of *synergy*,  = 1 of *additivity*, and <1 of *antagonism*.

## Results

### Hiltonol and GLA Synergize with R-848 to Promote the Maturation of CD14^+^ DDCs

**Figure 1:**
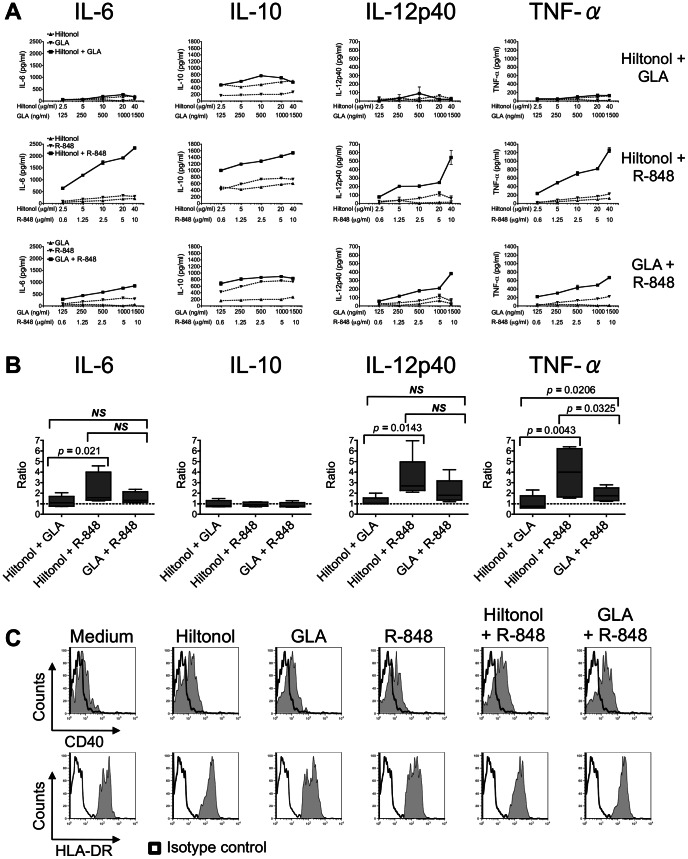
Selected TLR ligand combinations potently induce cytokine secretion by CD14^+^ DDCs. (A) Cytokine production after stimulation of CD14^+^ DDCs for 48 h with a range of concentrations (horizontal axes) of Hiltonol, GLA and R-848, or with combinations of these TLR ligands. The data are presented as means ± SEM of duplicates from one representative donor out of four independent experiments in which all 3 TLR combinations were tested at the same time. (B) Validation of potential synergistic effects of three TLR ligand combinations on cytokine expression. The ratio of the highest cytokine response elicited by the combined stimuli over the sum of the responses for the two individual agents at their constituent concentrations was calculated. A ratio >1 is indicative of synergy,  = 1 of additivity (displayed on charts as dashed lines), and <1 of antagonism. The data are presented as means ± SEM from 4–6 donors. (C) Phenotypic analysis of CD14^+^ DDCs stimulated with TLR ligand combinations. The expression of CD40 and HLA-DR was measured after 48 h. Black open histograms represent the isotype controls. One experiment representative of three is shown.

We have previously shown that Poly(I:C) and LPS synergize with R-848 to promote CD14^+^ DDCs to mature and then activate naïve B-cells in a co-culture system [14]. Our goal here was to assess whether the clinically approved adjuvants, Hiltonol and GLA, had similar properties when used in combinations. CD14^+^ DDCs were isolated after a 24 h culture of human skin samples. The various adjuvants were then applied, alone and together, over a wide range of concentrations, including ones normally sub-optimal for stimulating cytokine release. Compared to the individual agents, the Hiltonol plus R-848 and GLA plus R-848 combinations increased the secretion of several cytokines (IL-6, IL-10, IL-12p40 and TNF-α) to extents that ranged from additive to synergistic (Figure 1A). In contrast, the Hiltonol plus GLA combination was only slightly better than the individual agents (Figure 1A).

Of the three combinations tested, the rank order for potency at cytokine stimulation was Hiltonol plus R-848> GLA plus R-848> Hiltonol plus GLA. We assessed whether the enhancing effects of the various combinations were consistent with synergy or only additivity, as previously described [14]. The Hiltonol plus R-848 combination enhanced IL-6, IL-12p40 and TNF-α expression synergistically (ratios of >1), but IL-10 only additively (Figure 1B). Although the overall effects were less pronounced when GLA was combined with R-848, the trend was similar (Figure 1B).

In kinetic studies, the Hiltonol plus R-848 and GLA plus R-848 combinations triggered increases in IL-6, IL-10 and IL-12p40 expression that were apparent by 16 h and then sustained throughout the 48 h duration of the culture. In contrast, TNF-α expression increased more rapidly (by 4 h), but also more transiently (Figure S1). In accord with our previous findings [14], neither combination increased mRNA expression of the B-cell stimulatory cytokine BAFF over a 4–24 h period (Figure S1). The same two combinations modestly enhanced the phenotypic maturation of the CD14^+^ DDCs. However, they were only slightly superior to the individual agents at triggering CD40 and HLA-DR up-regulation (Figure 1C), an effect that was evident over a range of TLR ligand concentrations (Figure S2).

### Enhanced B-cell Stimulatory Capacity of Dual TLR Ligand-Stimulated CD14^+^ DDCs

We next studied how the TLR ligand-stimulated CD14^+^ DDCs affected the differentiation of naïve B-cells. Since soluble factor(s) released from CD14^+^ DDCs are involved in the stimulatory events [14], experiments were conducted by co-culturing CD14^+^ DDCs and naïve B-cells in the continuous presence of TLR ligands, together with IL-2 plus CD40L (to mimic T-cell help). The latter arrangement mimics the ménage-à-trois that exists between DCs, B-cells and T-cells [25]. For comparison, naïve B-cells were cultured without CD14^+^ DDCs, but in the presence of the same soluble agents.

**Figure 2:**
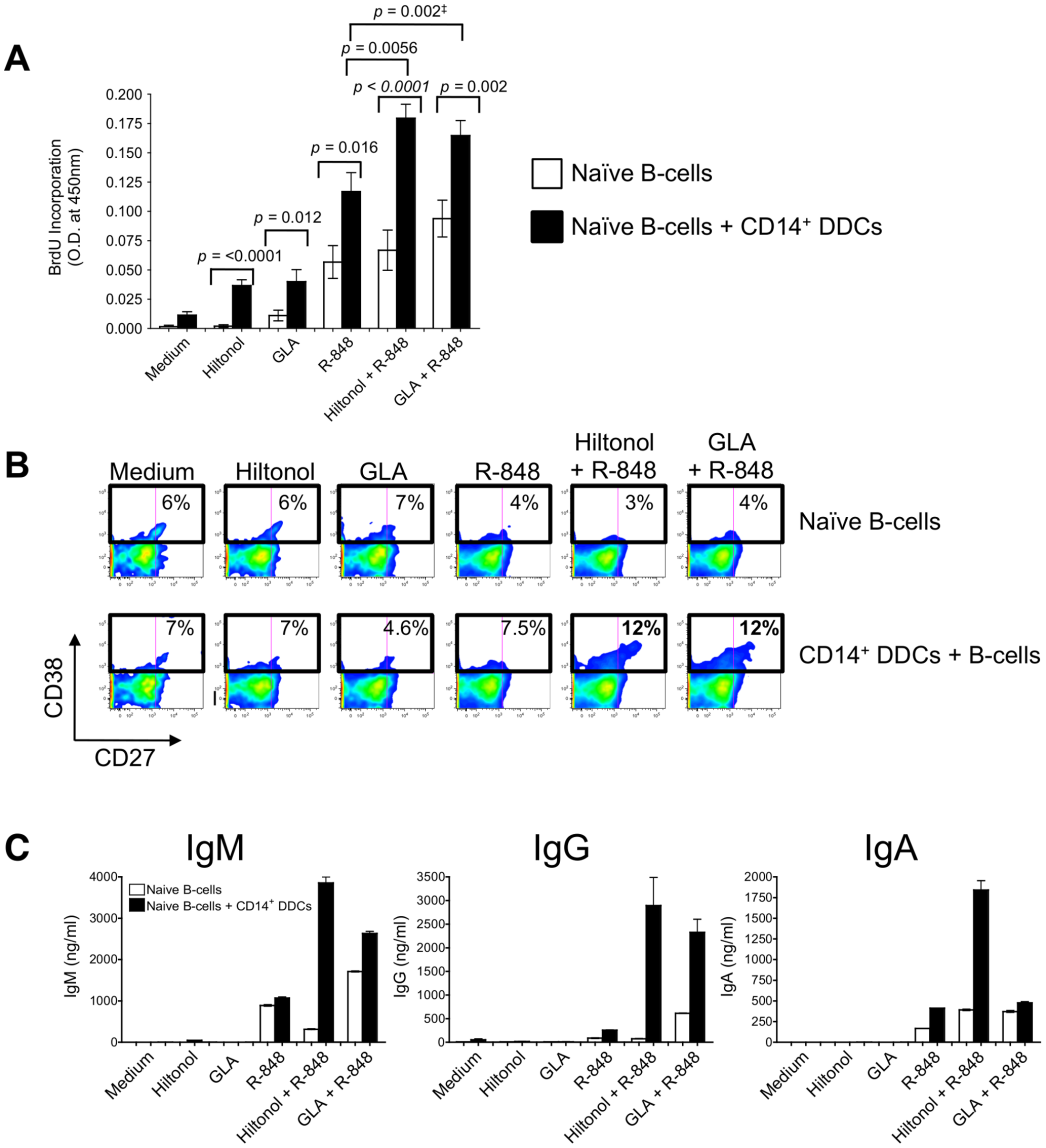
CD14^+^ DDCs stimulated with two TLR ligands have an increased capacity to induce naïve B-cells to proliferate and differentiate into Ig-secreting B-cells. (A) Proliferation of B-cells after stimulation with TLR ligands in the presence or absence of CD14^+^ DDCs, as measured by BrdU incorporation. The following concentrations of TLR ligands were used: Hiltonol (2.5 µg/ml), GLA (125 ng/ml) and R-848 (0.6 µg/ml). Naïve B-cells were isolated and cultured for 5 days with TLR ligands in the presence of CD40L and IL-2, and with or without CD14^+^ DDCs (DC: B-cell ratio of 1∶5). The bars represent mean O.D. values (± SEM) derived from triplicate samples from each of three donors. ^‡^Wilcoxon matched-pairs test. (B) Phenotypic analysis of B-cells after 7 days of stimulation with TLR ligands in the presence or absence of CD14^+^ DDCs (plus CD40L and IL-2). The numbers denote the percentage of CD38^+^ CD27^+^ B-cells. (C) Ig secretion in B-cell cultures stimulated for 14 days with the indicated TLR ligands, and CD40L plus IL-2, in the presence or absence of CD14^+^ DDCs. Ig secretion was quantified by ELISA. Data are presented as means ± SEM of duplicates and are representative of three independent experiments.

In the absence of CD14^+^ DDCs, R-848 stimulated B-cells to proliferate, a response that was further augmented by adding GLA but not Hiltonol (Figure 2A). When CD14^+^ DDCs were also present, B-cell proliferation was substantially greater under every tested condition. R-848 again enhanced the B-cell proliferative response, and the further inclusion of either GLA or Hiltonol had an additional potentiating effect (Figure 2A) (p = 0.002; Wilcoxon matched-pairs test) and p = 0.0056, respectively). Next, we addressed whether CD38 and CD27 were up-regulated on the B-cells under the same conditions. In co-cultures with control (unstimulated) CD14^+^ DDCs, 7% of the naïve B-cells acquired the CD38^+^ CD27^+^ phenotype. This percentage was increased only marginally, or not at all, when R-848, Hiltonol or GLA was included in the co-cultures (Figure 2B). However, when either Hiltonol or GLA was combined with R-848, 12% of the B-cells in the co-cultures now had the CD38^+^ CD27^+^ phenotype.

The stimulation of Ig secretion followed a generally similar pattern to the other markers of B-cell activation (Figure 2C). Thus, the highest levels of IgM, IgG and IgA release were observed when B-cells were co-cultured with CD14^+^ DDCs in the presence of Hiltonol plus R-848. The GLA plus R-848 combination had a lesser, although still marked, effect on IgM and IgG production in the co-cultures, but did not boost IgA secretion beyond the level seen with R-848 alone. Overall, combining Hiltonol with R-848 in the B-cell and CD14^+^ DDC co-cultures enhanced IgM, IgG and IgA production by 4-fold, ∼11-fold and ∼5-fold, compared to when R-848 was used by itself (Figure 2C).

Taken together, the above results show that in association with CD14^+^ DDCs, naïve B-cells are activated efficiently by Hiltonol plus R-848, and to a lesser extent by GLA plus R-848. The stimulated B-cells both proliferate and differentiate to CD38-expressing B-cells that secrete immunoglobulins.

### CD14^+^ DDCs have Enhanced Th1-polarizing Capacity when Stimulated with Selected TLR Ligand Combinations

**Figure 3:**
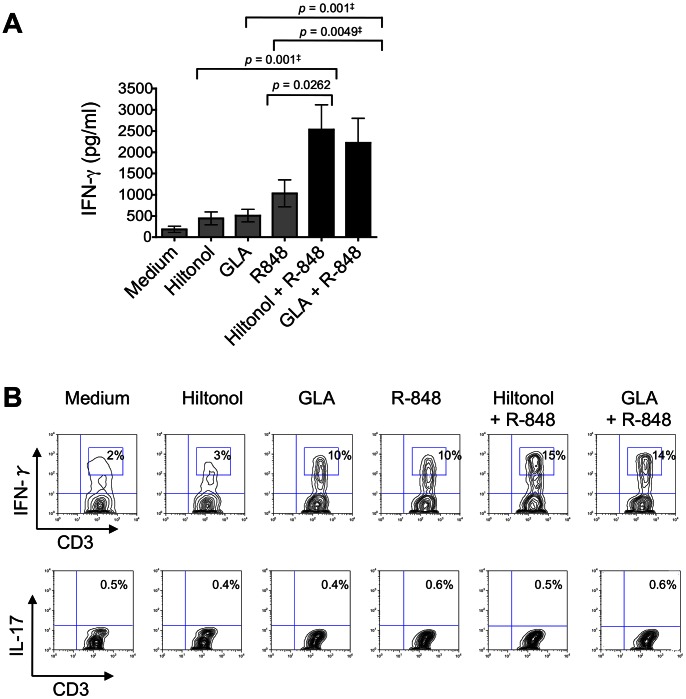
Dual TLR ligand-stimulated CD14^+^ DDCs enhance Th1 but not Th17-cell differentiation. (A) Cytokine secretion by responder allogeneic CD4^+^ T-cells after culture for 6 days with TLR ligand-stimulated CD14^+^ DDCs. The CD14^+^ DDCs were stimulated for 24 h with Hiltonol, GLA, R-848, Hiltonol plus R-848 or GLA plus R-848. Allogeneic naïve (CD27^+^ CD45RO^−^) CD4^+^ T-cells from a healthy donor were then added to the DDCs in 96-well flat-bottomed plates. Supernatants were collected on day 6 of the co-culture and screened for IFN-γ by ELISA. The data are presented as means ± SEM of duplicate samples from three independent experiments. ^‡^Wilcoxon matched-pairs test. (B) Intracellular cytokine expression by responder allogeneic CD4^+^ T-cells after 6 days of co-culture. T-cells were tested for their capacity to secrete IFN-γ and IL-17 after re-stimulation for 6 h with PMA and ionomycin in the presence of brefeldin. The numbers in the boxed regions indicate the percentage of CD4^+^ T-cells expressing intracellular IFN-γ or IL-17. The data shown are from one of three experiments performed.

We next evaluated how TLR ligation affected the Th-priming capabilities of CD14^+^ DDCs. To do so, we stimulated CD14^+^ DDCs with single TLR ligands or the combinations described above, and tested their capacity to prime naïve allogeneic CD4^+^ T-cells from several donors. When CD14^+^ DDCs were stimulated with R-848 plus either Hiltonol or GLA, they induced Th1-cell polarization (IFN-γ secretion and the proportion of IFN-γ^+^ CD4^+^ T-cells) to a greater extent than when any single TLR ligand was used (Figure 3A and B, upper panel). None of the tested conditions triggered the differentiation of Th17 cells. Thus, after re-stimulation with PMA and ionomycin, <0.5% of T-cells stained positive for IL-17 (Figure 3B, lower panel). We also screened co-culture supernatants for IL-21, a cytokine expressed by CD4^+^ T follicular helper cells. None of the tested conditions triggered the differentiation of IL-21^+^ CD4^+^ T-cells. Thus, IL-21 was undetectable in (undiluted) supernatants from all donors tested (data not shown).

### HIV-1 gp120 does not Compromise the Capacity of Selected TLR Ligand Combinations to Activate CD14^+^ DDCs

**Figure 4:**
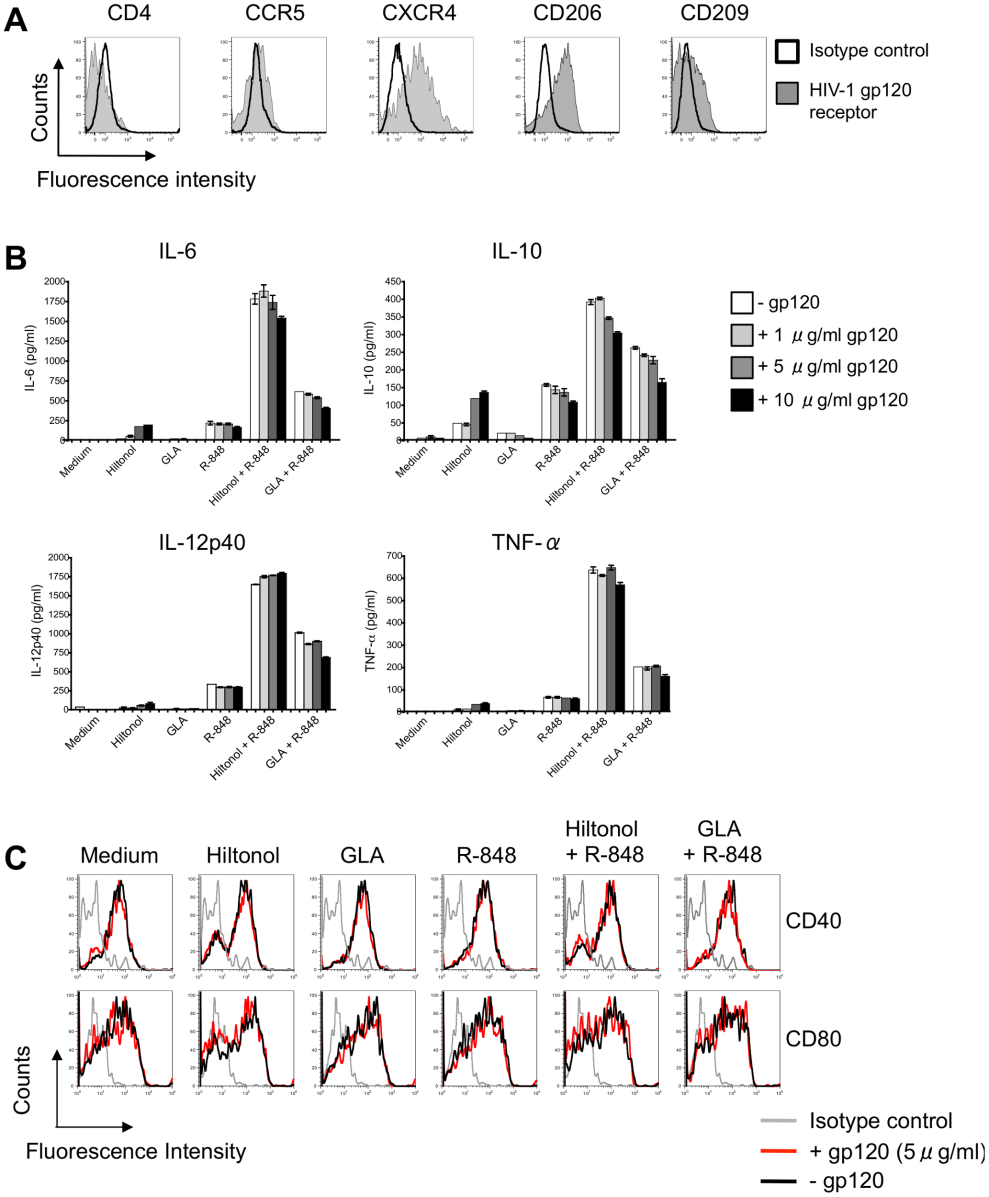
HIV-1 gp120 does not compromise the capacity of selected TLR ligand combinations to activate CD14^+^ DDCs. (A) Expression of gp120-binding receptors by CD14^+^ DDCs. Migratory cells were stained for MAbs specific for the following gp120-binding receptors: CD4, CCR5 (CD195), CXCR4 (CD184), CD206 and CD209, after gating on CD14^+^ HLA-DR^+^ DDCs. The data are representative of three independent experiments. The black open histograms represent the isotype controls. (B) Cytokine secretion by CD14^+^ DDCs in response to TLR ligand(s) and gp120. CD14^+^ DDCs were exposed to different concentrations of JR-FL gp120 (1–10 µg/ml) for 1 h at 37°C before adding TLR ligand(s) for 48 h. The following concentrations were used: Hiltonol (10 µg/ml), GLA (500 ng/ml) and R-848 (2.5 µg/ml). IL-6, IL-10, IL-12p40 and TNF-α were quantified in supernatants by ELISA. The data are representative of three independent experiments and are presented as means ± SEM of duplicate samples. (C) Phenotype of CD14^+^ DDCs after stimulation with TLR ligand(s) in the presence or absence of HIV-1 gp120. The data are from one of three experiments performed. Gray histograms: isotype control; Open black histograms: CD14^+^ DDCs stimulated in the absence of gp120; Open red histograms: CD14^+^ DDCs stimulated in the presence of 5 µg/ml JR-FL gp120.

HIV-1 gp120 can exert suppressive effects on a variety of immune cells *in vitro*, including monocyte-derived DCs [17] and plasmacytoid DCs [18], [19] and can under some circumstances suppress immune responses *in vivo* [20]–[22], [24]. We therefore assessed whether gp120 could interfere with the ability of the above TLR ligand combinations to activate CD14^+^ DDCs. Among potential gp120-binding receptors, the mannose macrophage receptor (CD206), DC-SIGN (CD209) and CXCR4 (CD184) were expressed strongly on migratory CD14^+^ DDCs, but CD4 and CCR5 (CD195) only weakly (Figure 4A).

A similar preparation of DDCs was then exposed to monomeric gp120 (JR-FL, clade B) for 1 h before the addition of TLR ligands, alone or in combination. Even a high concentration (10 µg/ml) of gp120 had only a modest suppressive effect upon TLR ligand-triggered cytokine secretion (Figure 4B) and maturation marker (CD40 and CD80) up-regulation (Figure 4C). Of note is that same gp120 stock significantly suppressed the release of IFN-α from CpG-ODN 2006 (type B CpG)-stimulated plasmacytoid DCs (data not shown) [19].

### Selected TLR Ligand Combinations Activate Multiple Cutaneous DC Subsets

**Figure 5:**
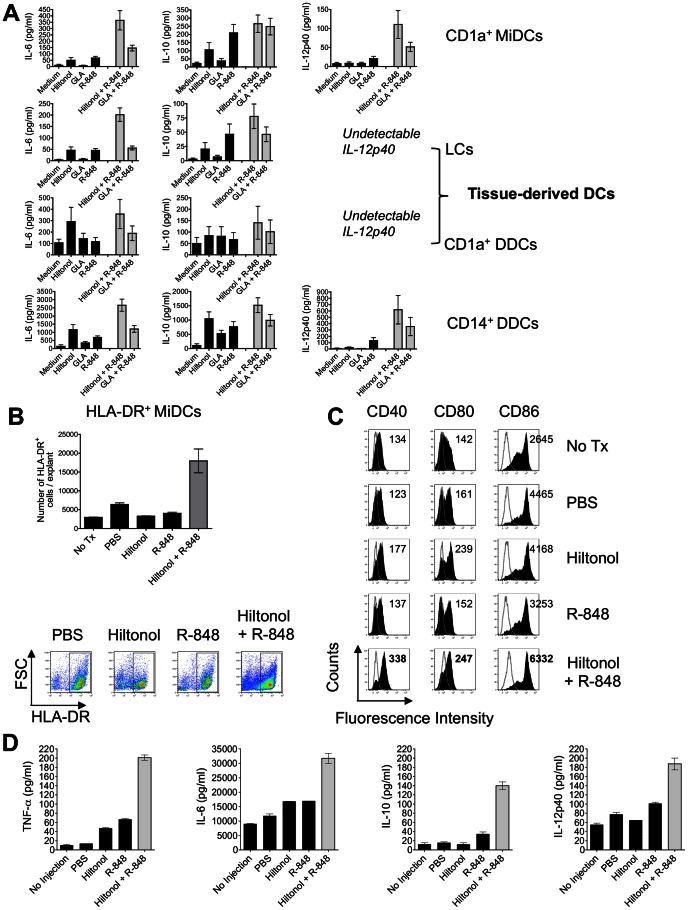
The Hiltonol plus R-848 combination potently activates multiple skin DC subsets. (A) The Hiltonol plus R-848 combination enhances cytokine secretion from multiple DC subsets after stimulation with selected TLR ligand(s). CD1a^+^ MiDCs (n = 5 donors), tissue-derived LCs (n = 4 donors) and CD1a^+^ DDCs (n = 3 donors) were stimulated with TLR ligand(s) for 48 h (∼2×10^4^/condition) before cytokine concentrations were quantified by ELISA. For comparison, data derived using CD14^+^ DDCs are also displayed (n = 7 donors). The data are presented as means ± SEM. (B) Enhanced migration of MiDCs after microinjection with Hiltonol plus R-848. MiDCs were quantified by flow cytometry after gating on FSC^hi^ SSC^hi^ HLA-DR^+^ cells. (C) Phenotypic analysis of MiDCs following microinjection of skin explants with TLR ligand(s). Skin explants were injected with PBS, Hiltonol, R-848, or Hiltonol plus R-848. Explants were cultured for 48 h and compared to explants receiving no treatment (no Tx). Migratory DCs were identified as large (FSC^hi^ SSC^hi^), HLA-DR^+^ cells and analyzed for CD40, CD80 and CD86 expression. Mean fluorescence intensity (MFI; geometric mean) are displayed on histograms. Gray open histograms represent isotype controls. (D) Cytokine expression in supernatants from skin explants following microinjection with TLR ligand(s). Cell-free supernatants were collected 48 h after injection and their IL-6, IL-10, TNF-α and IL-12p40 content was quantified by ELISA.

Although we confirmed that our selected adjuvant combinations strongly activate CD14^+^ DDCs, they may also have a stimulatory effect upon other cutaneous DC subsets, since there is overlap in TLR expression. For example, both tissue-derived LCs and CD1a^+^ DDCs endogenously express TLRs 3 and 7 (Figure S3) [26]. We observed that both the Hiltonol plus R-848 and GLA plus R-848 combinations were superior to the individual ligands at triggering cytokine secretion from CD1a^+^ MiDCs (Figure 5A). Combining Hiltonol with R-848 enhanced the production of both IL-6 and IL-12p40 by CD1a^+^ MiDCs synergistically, but IL-10 expression was increased only modestly (Figure S4). The GLA plus R-848 combination also had synergistic effects on IL-6 and IL-12p40 production, although not to the same extent as seen with Hiltonol plus R-848 (Figure S4).

CD1a^+^ MiDCs are heterogeneous, with both emigrating CD1a^+^ epidermal LCs and CD1a^+^ DDCs present. When these two DC subsets were purified (directly from skin) and stimulated with Hiltonol plus R-848, an enhancing effect upon cytokine secretion was observed compared to treatment with individual agents (Figure 5A), in line with the expression of both TLRs 3 and 7 (Figure S3). CD1a^+^ DDCs also express TLRs 4 and 8 (but at lower levels than CD14^+^ dermal-derived DCs; Figure S3) and a modest enhancing effect upon cytokine secretion was observed when GLA and R-848 were combined (Figure 5A). In contrast, the GLA plus R-848 combination had no enhancing effect relative to individual agents on LCs (Figure 5A), likely due to the low expression of TLR4 (Figure S3).

Since the Hiltonol plus R-848 combination was the most potent at activating not only CD14^+^ DDCs (Figure 1 and Figure 5A) but also LCs and CD1a^+^ DDCs (Figure 5A), we performed intradermal injection studies to assess its effects *in situ*. Skin explants were injected intradermally with PBS, Hiltonol, R-848, or Hiltonol plus R-848, using ultrafine needles, and migratory cells and supernatants were collected 48 h later. The egress of migratory DCs (HLA-DR^++^) was greater when Hiltonol was co-injected with R-848 than when either individual agent, or PBS, was administered (Figure 5B). The absolute number of emigrating DCs increased after co-injection of Hiltonol plus R-848, but there was little change in the relative proportion of the CD14^+^ and CD14^−^ DC subsets, compared to when PBS or the individual agents were injected (data not shown). The injection of R-848 into the skin explants up-regulated CD80 and CD86 on MiDCs to no greater extent than when PBS was used (Figure 5C). In contrast, DCs emigrating from Hiltonol-injected explants had a more mature phenotype, an outcome boosted when Hiltonol was combined with R-848 (Figure 5C). Of note is that both CD14^+^ DDCs and CD14^−^ MiDCs expressed higher levels of both CD40 and CD86 after injection with Hiltonol plus R-848 than when the individual TLR ligands were used (data not shown). Morever, the combination triggered a greater release of cytokines (IL-6, IL-10, IL-12p40 and TNF-α) into the skin biopsy supernatants than either TLR ligand by itself (Figure 5D). Mixing Hiltonol with R-848 therefore represents an attractive adjuvant formulation for activating multiple DC subsets, including CD14^+^ DDCs.

## Discussion

Traditionally, vaccines are administered by the subcutaneous or intramuscular routes, but there is accumulating evidence that targeting the skin directly could be particularly advantageous [10]. Thus, intradermal immunization was superior to subcutaneous or transcutaneous delivery for inducing polyvalent responses to HIV-1 Gag [27]. Selectively targeting distinct DC subsets may be desirable when a specific immune response is required [11]. For example, a strong CTL response would be best achieved via epidermal LCs, whereas targeting CD14^+^ DDCs could be advantageous for inducing good humoral responses [13].

Our goals here were: first, to determine if the Hiltonol and GLA adjuvants, both of which are in clinical development, can activate CD14^+^ DDCs more strongly when combined with R-848 than they do alone; second, to assess whether these TLR ligand combinations can further augment the B-cell and CD4^+^ T-cell stimulatory capacity of CD14^+^ DDCs; third, to see if the chosen combinations were compromised by the addition of HIV-1 gp120, which can exert immunosuppressive effects under some conditions. The overarching aim was to determine whether combinations of the above TLR ligands might be suitable adjuvants for delivering HIV-1 Env-based vaccines to humans by the intradermal route.

Cytokine secretion was strongly enhanced when CD14^+^ DDCs were stimulated with the Hiltonol plus R-848 or GLA plus R-848 combinations, compared to when the individual TLR ligands were tested. This outcome is consistent with our earlier study using different TLR ligands [14]. Furthermore, CD14^+^ DDCs exposed to either combination potently stimulated naïve B-cells to proliferate and differentiate into CD38^+^ B-cells that secreted high levels of immunoglobulins. These B-cell responses to TLR-ligand activated CD14^+^ DDCs were superior to when naïve B-cells were stimulated directly with the same agents (i.e., without CD14^+^ DDCs). The GLA plus R-848 combination was also better than the individual components at directly activating naïve B-cells. While human naïve B-cells endogenously express low levels of mRNA for TLR7/8, they have not been reported to express high levels of TLR4, the target for GLA. Of note is that strong Ab responses may require the direct triggering of TLRs on both B-cells and DCs, based on observations in mice vaccinated with nanoparticles containing antigen together with TLR4 (MPL) and TLR7 (R-837) ligands [28].

HIV-1 gp120 can bind to many immune-system molecules, including CD4, CCR5, CXCR4 and MCLRs, thereby potentially interfering with key components of the immune system [16]. Suppressive effects of gp120 on diverse functions of a number of immune cells, including plasmacytoid DCs and monocyte-derived DCs, have been observed *in vitro* [17]–[19], and gp120 can also suppress immune responses *in vivo* [20]–[22], [24]. Given that Env vaccines are often delivered in considerable amounts (several hundred µg) to local sites, it is possible that the receptor-binding properties of these proteins could impair their immunogenicity [16]. We therefore assessed whether monomeric gp120 might impede the activation of CD14^+^ DDCs by our selected TLR ligand combinations. Although CD14^+^ DDCs express a number of gp120-binding receptors, including MCLRs, gp120 had no significant immunosuppressive effects on these cells. Thus, DDCs stimulated with our selected adjuvant combinations in the presence or absence of gp120 secreted almost equivalent levels of cytokines.

Activating multiple DC subsets via TLR engagement, e.g., by combining Hiltonol or GLA with R-848, might be particularly advantageous given the value of having polyvalent responses to vaccines. Thus, the involvement of several TLRs (specifically TLRs 2, 7, 8 and 9) on different DC subsets underlies the success of the yellow-fever (YF-17D) vaccine [29]. Transcriptional analysis of blood samples from volunteers injected subcutaneously with Hiltonol showed that genes involved in innate immune pathways were up-regulated to an extent comparable to that seen in recipients of the YF-17D vaccine [30]; new studies but now incorporating R-848 and GLA seem warranted.

We also investigated whether our chosen adjuvant combinations activated other cutaneous DC subsets. Both combinations were superior to the individual components at stimulating cytokine secretion from CD1a^+^ MiDCs, with Hiltonol plus R-848 being the more effective. The latter combination also enhanced cytokine secretion from tissue-derived LCs and CD1a^+^ DDCs in cell culture, and it triggered the migration of DCs with a mature, cytokine-secreting phenotype when injected into skin explants. These effects of the Hiltonol plus R-848 combination were greater than when the individual agents were injected. Given the inherent role of LCs in priming naïve CD8^+^ T-cells into CTLs [13], [31], enhanced CTL responses could be expected after intradermal injection with antigen in the presence of Hiltonol plus R-848. Indeed, antigen-specific CTLs were strongly activated *in vitro* when peptide-pulsed monocyte-derived DCs were stimulated with Hiltonol plus R-848 [32].

In summary, we have shown that two clinically relevant adjuvant combinations (Hiltonol plus R-848 and GLA plus R-848) substantially increased the endogenous capacity of CD14^+^ DDCs to differentiate naïve B-cells into CD38^+^ CD27^+^ plasmablast-like B-cells capable of secreting high levels of immunoglobulins. The dual TLR-stimulated CD14^+^ DDCs also have strongly augmented Th1-polarizing capacity. These boosting actions of the adjuvants on DDC functions were not compromised by HIV-1 gp120. Targeting dermal CD14^+^ DDCs and LCs with TLR ligand combinations, particularly Hiltonol plus R-848, may be a way to induce strong humoral immune and cellular responses simultaneously. Such an outcome is relevant to the design of various vaccines including, but not limited to, ones containing HIV-1 Env.

## Supporting Information

Figure S1 **Kinetics of cytokine expression after stimulation of CD14^+^ DDCs with selected TLR ligand combinations.** The following concentrations of TLR ligands were used: Hiltonol (10 µg/ml), GLA (500 ng/ml) and R-848 (2.5 µg/ml). (A) Kinetics of IL-6, IL-10, IL-12p40 and TNF-α secretion. Data are presented as means ± SEM of duplicate samples from one representative experiment of three performed. (B) Quantitative RT-PCR analysis of BAFF mRNA expression in CD14^+^ DDCs stimulated for 1–24 h with selected TLR ligands. The housekeeping gene GAPDH was used for normalizing mRNA recovery.
(TIF)

Figure S2 **Enhanced phenotypic maturation of CD14^+^ DDCs is evident after stimulation with different TLR ligand combinations.** CD40 expression was measured after 48 h. The black open histograms represent the isotype controls, the red open histograms unstimulated CD14^+^ DDCs (“baseline”, where MFI = 302) and the closed black histograms CD14^+^ DDCs stimulated with the indicated TLR ligand(s). The numbers in the histograms denote (geometric) MFI.
(TIF)

Figure S3 **Differential expression of TLR mRNA in DC subsets isolated directly from skin.** (A) Highly purified DDCs (CD1a^+^ and CD14^+^ subsets) and LCs were isolated by FACS from dermal and epidermal tissues, respectively. The gating strategy for each DC subset is shown. Successive gating on live (7-AAD^−^) (R2), CD45^+^ (R3) and HLA-DR^+^ (R4) cells was performed in each case, followed by additional gating: ***Upper panel (dermal digests):*** CD1a^+^ DDCs were isolated after gating on CD1a^+^ CD14^−^ cells (R5). For comparison, CD14^+^ DDCs were also isolated (CD14^+^ CD1a^−^ (R6)), after further gating on CD1c^+^ SSC^lo^ cells (R7) (not shown). ***Lower panel (epidermal digests):*** LCs were isolated after gating on CD1a^+^ CD207^+^ cells (R5). (B) Quantitative expression of TLRs 3, 4, 7 and 8 mRNA in DC subsets isolated from skin. cDNA samples from three different donors were tested (in triplicate) for each gene of interest and normalized against GAPDH by Taqman® Real-time RT-PCR.
(TIF)

Figure S4 **Selected TLR ligand combinations synergistically enhance expression of IL-6 and IL-12p40 by CD1a^+^ MiDCs.** Validation of potential synergistic effects of Hiltonol plus R-848 and GLA plus R-848 on cytokine expression by CD1a^+^ MiDCs. The ratio of the highest cytokine response elicited by the combined stimuli over the sum of the responses for the two individual agents at their constituent concentrations was calculated. A ratio >1 is indicative of synergy,  = 1 of additivity (displayed on charts as dashed lines), and <1 of antagonism. The data are presented as means ± SEM. Samples from five different donors were individually tested using each TLR ligand combination.
(TIF)

## References

[1] Coffman RL, Sher A, Seder RA (2010) Vaccine adjuvants: putting innate immunity to work. Immunity 33: 492–503. 21029960 10.1016/j.immuni.2010.10.002 PMC3420356

[2] Banchereau J, Steinman RM (1998) Dendritic cells and the control of immunity. Nature 392: 245–252. 9521319 10.1038/32588

[3] Pashine A, Valiante NM, Ulmer JB (2005) Targeting the innate immune response with improved vaccine adjuvants. Nat Med 11: S63–68. 15812492 10.1038/nm1210

[4] Napolitani G, Rinaldi A, Bertoni F, Sallusto F, Lanzavecchia A (2005) Selected Toll-like receptor agonist combinations synergistically trigger a T helper type 1-polarizing program in dendritic cells. Nat Immunol 6: 769–776. 15995707 10.1038/ni1223 PMC3760217

[5] Shen H, Tesar BM, Walker WE, Goldstein DR (2008) Dual signaling of MyD88 and TRIF is critical for maximal TLR4-induced dendritic cell maturation. J Immunol 181: 1849–1858. 18641322 10.4049/jimmunol.181.3.1849 PMC2507878

[6] Reed SG, Bertholet S, Coler RN, Friede M (2009) New horizons in adjuvants for vaccine development. Trends Immunol 30: 23–32. 19059004 10.1016/j.it.2008.09.006

[7] Coler RN, Bertholet S, Moutaftsi M, Guderian JA, Windish HP, et al (2011) Development and characterization of synthetic glucopyranosyl lipid adjuvant system as a vaccine adjuvant. PLoS One 6: e16333. 21298114 10.1371/journal.pone.0016333 PMC3027669

[8] Coler RN, Baldwin SL, Shaverdian N, Bertholet S, Reed SJ, et al (2010) A synthetic adjuvant to enhance and expand immune responses to influenza vaccines. PLoS One 5: e13677. 21060869 10.1371/journal.pone.0013677 PMC2965144

[9] Teunissen MB, Haniffa M, Collin MP (2012) Insight into the immunobiology of human skin and functional specialization of skin dendritic cell subsets to innovate intradermal vaccination design. Curr Top Microbiol Immunol 351: 25–76. 21833835 10.1007/82_2011_169

[10] Combadiere B, Liard C (2011) Transcutaneous and intradermal vaccination. Hum Vaccin 7: 811–827. 21817854 10.4161/hv.7.8.16274

[11] Klechevsky E, Liu M, Morita R, Banchereau R, Thompson-Snipes L, et al (2009) Understanding human myeloid dendritic cell subsets for the rational design of novel vaccines. Hum Immunol 70: 281–288. 19236899 10.1016/j.humimm.2009.02.004 PMC2674516

[12] Caux C, Massacrier C, Vanbervliet B, Dubois B, Durand I, et al (1997) CD34+ hematopoietic progenitors from human cord blood differentiate along two independent dendritic cell pathways in response to granulocyte-macrophage colony-stimulating factor plus tumor necrosis factor alpha: II. Functional analysis. Blood 90: 1458–1470. 9269763

[13] Klechevsky E, Morita R, Liu M, Cao Y, Coquery S, et al (2008) Functional specializations of human epidermal Langerhans cells and CD14+ dermal dendritic cells. Immunity 29: 497–510. 18789730 10.1016/j.immuni.2008.07.013 PMC2688399

[14] Matthews K, Chung NP, Klasse PJ, Moore JP, Sanders RW (2012) Potent Induction of Antibody-Secreting B Cells by Human Dermal-Derived CD14+ Dendritic Cells Triggered by Dual TLR Ligation. J Immunol. 10.4049/jimmunol.1200601 PMC3951118 23162132

[15] Pantophlet R, Burton DR (2006) GP120: target for neutralizing HIV-1 antibodies. Annu Rev Immunol 24: 739–769. 16551265 10.1146/annurev.immunol.24.021605.090557

[16] Klasse PJ, Sanders RW, Cerutti A, Moore JP (2012) How Can HIV-Type-1-Env Immunogenicity Be Improved to Facilitate Antibody-Based Vaccine Development? AIDS Res Hum Retroviruses 28: 1–15. 21495876 10.1089/aid.2011.0053 PMC3251839

[17] Shan M, Klasse PJ, Banerjee K, Dey AK, Iyer SP, et al (2007) HIV-1 gp120 mannoses induce immunosuppressive responses from dendritic cells. PLoS Pathog 3: e169. 17983270 10.1371/journal.ppat.0030169 PMC2048530

[18] Martinelli E, Cicala C, Van Ryk D, Goode DJ, Macleod K, et al (2007) HIV-1 gp120 inhibits TLR9-mediated activation and IFN-{alpha} secretion in plasmacytoid dendritic cells. Proc Natl Acad Sci U S A 104: 3396–3401. 17360657 10.1073/pnas.0611353104 PMC1805537

[19] Chung NP, Matthews K, Klasse PJ, Sanders RW, Moore JP (2012) HIV-1 gp120 Impairs the Induction of B Cell Responses by TLR9-Activated Plasmacytoid Dendritic Cells. J Immunol. 10.4049/jimmunol.1201905 PMC3504132 23100517

[20] van Montfort T, Sanders RW (2012) Optimizing cellular immunity against HIV-1 Gag and preventing suppression by HIV-1 gp120. Expert Rev Vaccines 11: 1175–1177. 23176650 10.1586/erv.12.102

[21] Toapanta FR, Craigo JK, Montelaro RC, Ross TM (2007) Reduction of anti-HIV-1 Gag immune responses during co-immunization: immune interference by the HIV-1 envelope. Curr HIV Res 5: 199–209. 17346134 10.2174/157016207780077057

[22] Hovav AH, Santosuosso M, Bivas-Benita M, Plair A, Cheng A, et al (2009) X4 human immunodeficiency virus type 1 gp120 down-modulates expression and immunogenicity of codelivered antigens. J Virol 83: 10941–10950. 19692474 10.1128/JVI.00394-09 PMC2772807

[23] Banerjee K, Andjelic S, Klasse PJ, Kang Y, Sanders RW, et al (2009) Enzymatic removal of mannose moieties can increase the immune response to HIV-1 gp120 in vivo. Virology 389: 108–121. 19410272 10.1016/j.virol.2009.04.001 PMC2743082

[24] Banerjee K, Michael E, Eggink D, van Montfort T, Lasnik AB, et al (2012) Occluding the mannose moieties on human immunodeficiency virus type 1 gp120 with griffithsin improves the antibody responses to both proteins in mice. AIDS Res Hum Retroviruses 28: 206–214. 21793733 10.1089/aid.2011.0101 PMC3275927

[25] Ueno H, Schmitt N, Palucka AK, Banchereau J (2010) Dendritic cells and humoral immunity in humans. Immunol Cell Biol 88: 376–380. 20309010 10.1038/icb.2010.28 PMC2865578

[26] van der Aar AM, Sylva-Steenland RM, Bos JD, Kapsenberg ML, de Jong EC, et al (2007) Loss of TLR2, TLR4, and TLR5 on Langerhans cells abolishes bacterial recognition. J Immunol 178: 1986–1990. 17277101 10.4049/jimmunol.178.4.1986

[27] Liard C, Munier S, Arias M, Joulin-Giet A, Bonduelle O, et al (2011) Targeting of HIV-p24 particle-based vaccine into differential skin layers induces distinct arms of the immune responses. Vaccine 29: 6379–6391. 21554912 10.1016/j.vaccine.2011.04.080

[28] Kasturi SP, Skountzou I, Albrecht RA, Koutsonanos D, Hua T, et al (2011) Programming the magnitude and persistence of antibody responses with innate immunity. Nature 470: 543–547. 21350488 10.1038/nature09737 PMC3057367

[29] Querec T, Bennouna S, Alkan S, Laouar Y, Gorden K, et al (2006) Yellow fever vaccine YF-17D activates multiple dendritic cell subsets via TLR2, 7, 8, and 9 to stimulate polyvalent immunity. J Exp Med 203: 413–424. 16461338 10.1084/jem.20051720 PMC2118210

[30] Caskey M, Lefebvre F, Filali-Mouhim A, Cameron MJ, Goulet JP, et al (2011) Synthetic double-stranded RNA induces innate immune responses similar to a live viral vaccine in humans. J Exp Med 208: 2357–2366. 22065672 10.1084/jem.20111171 PMC3256967

[31] Banchereau J, Thompson-Snipes L, Zurawski S, Blanck JP, Cao Y, et al.. (2012) The differential production of cytokines by human Langerhans Cells and dermal CD14+ DCs controls CTL priming. Blood. 10.1182/blood-2011-08-371245 PMC3382933 22535664

[32] Bogunovic D, Manches O, Godefroy E, Yewdall A, Gallois A, et al (2011) TLR4 engagement during TLR3-induced proinflammatory signaling in dendritic cells promotes IL-10-mediated suppression of antitumor immunity. Cancer Res 71: 5467–5476. 21730023 10.1158/0008-5472.CAN-10-3988 PMC3156386

