# Supplementary figures and images for: Clinical Adjuvant Combinations Stimulate Potent B-Cell Responses In Vitro by Activating Dermal Dendritic Cells
Source: PLoS One. 2013 May 20;8(5):e63785. doi: 10.1371/journal.pone.0063785 (PMC3659025; doi:10.1371/journal.pone.0063785)

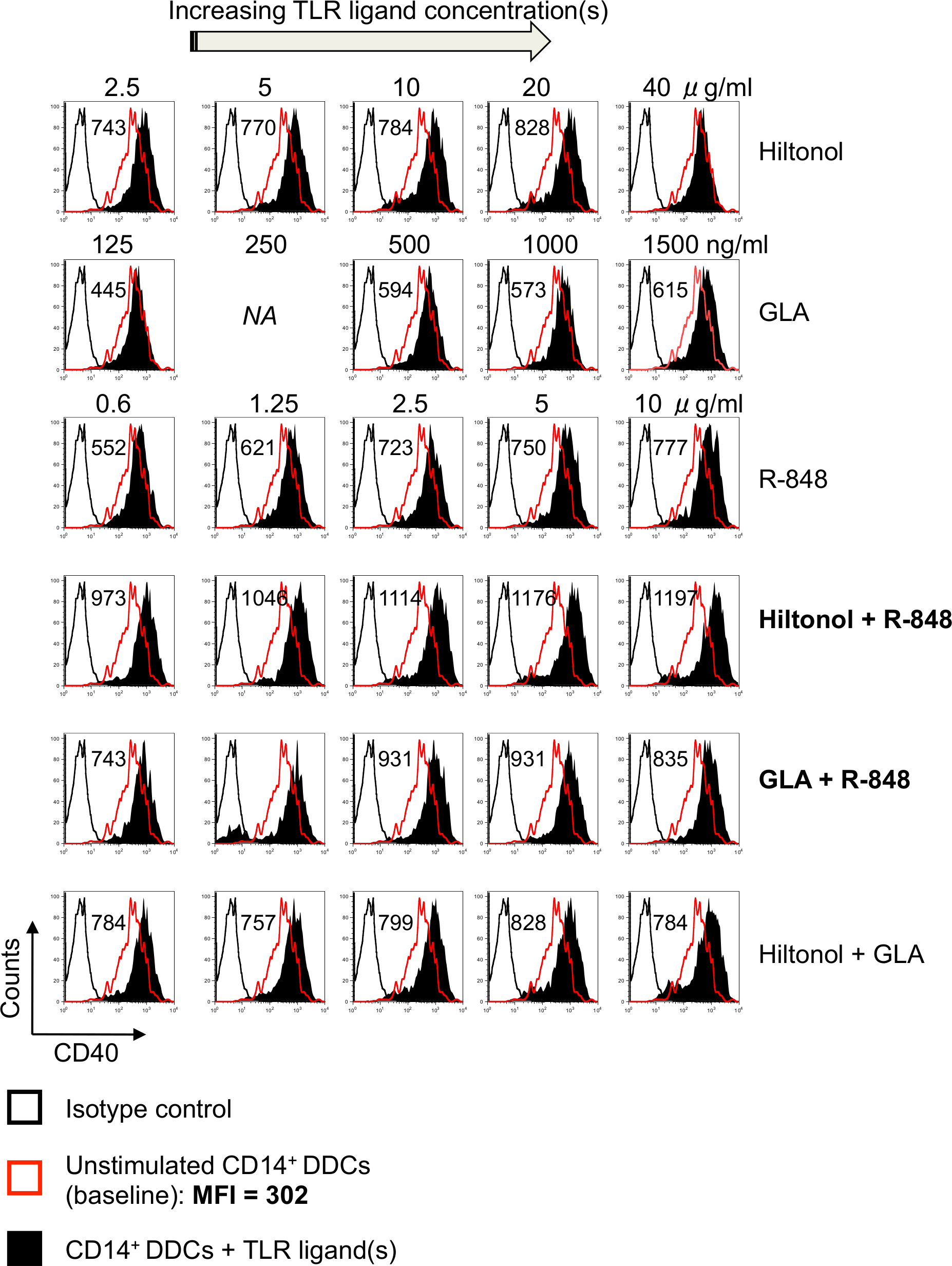

Supplement: Figure S2 — Enhanced phenotypic maturation of CD14+ DDCs is evident after stimulation with different TLR ligand combinations. CD40 expression was measured after 48 h. The black open histograms represent the isotype controls, the red open histograms unstimulated CD14+ DDCs (“baseline”, where MFI = 302) and the closed black histograms CD14+ DDCs stimulated with the indicated TLR ligand(s). The numbers in the histograms denote (geometric) MFI. (TIF) [file pone.0063785.s002.tif]

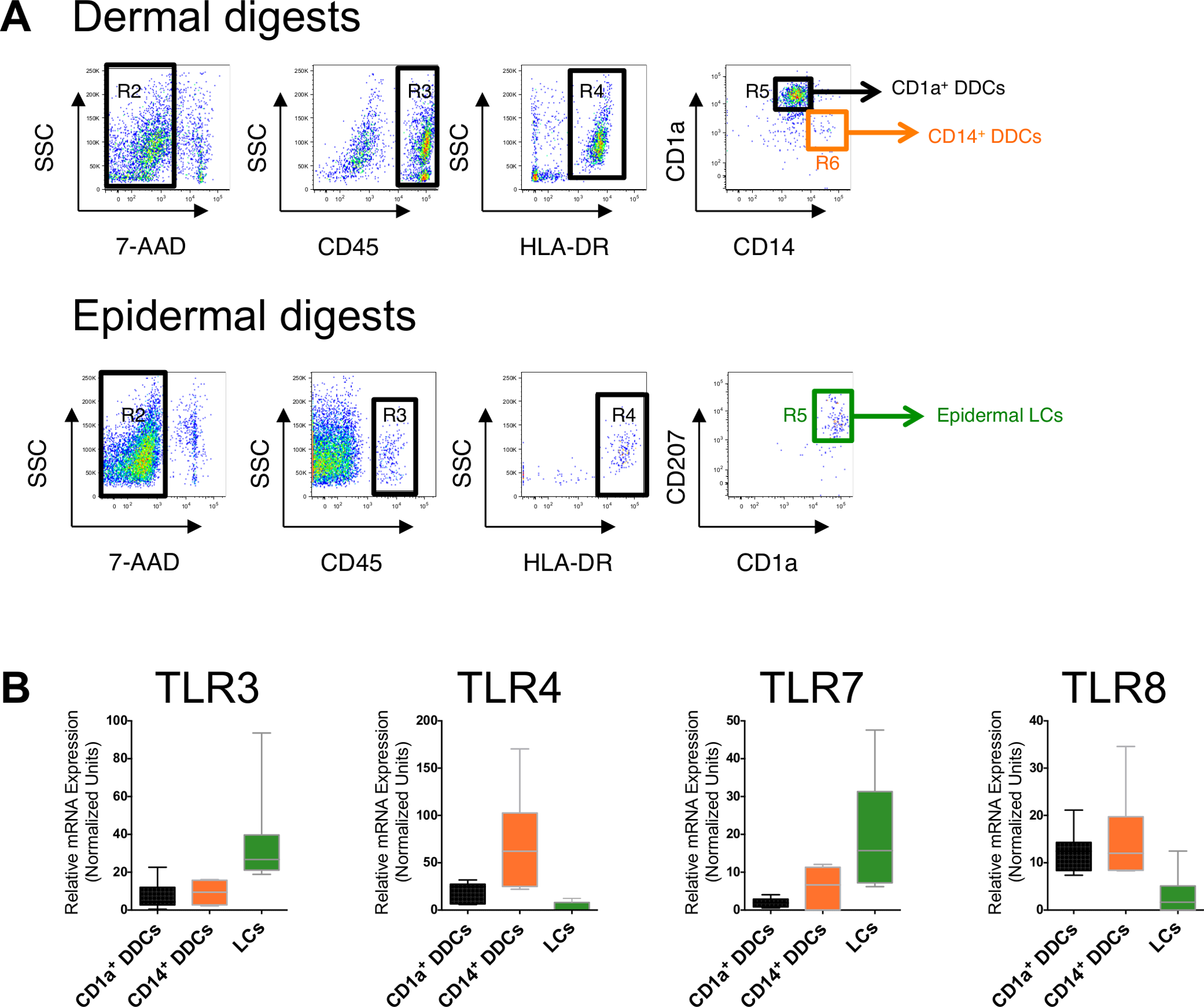

Supplement: Figure S3 — Differential expression of TLR mRNA in DC subsets isolated directly from skin. (A) Highly purified DDCs (CD1a+ and CD14+ subsets) and LCs were isolated by FACS from dermal and epidermal tissues, respectively. The gating strategy for each DC subset is shown. Successive gating on live (7-AAD−) (R2), CD45+ (R3) and HLA-DR+ (R4) cells was performed in each case, followed by additional gating: Upper panel (dermal digests): CD1a+ DDCs were isolated after gating on CD1a+ CD14− cells (R5). For comparison, CD14+ DDCs were also isolated (CD14+ CD1a− (R6)), after further gating on CD1c+ SSClo cells (R7) (not shown). Lower panel (epidermal digests): LCs were isolated after gating on CD1a+ CD207+ cells (R5). (B) Quantitative expression of TLRs 3, 4, 7 and 8 mRNA in DC subsets isolated from skin. cDNA samples from three different donors were tested (in triplicate) for each gene of interest and normalized against GAPDH by Taqman® Real-time RT-PCR. (TIF) [file pone.0063785.s003.tif]
